# Exploring member data for Community Supported Agriculture (CSA) in California: Comparisons of former and current CSA members

**DOI:** 10.1016/j.dib.2018.11.045

**Published:** 2018-11-13

**Authors:** Ryan E. Galt, Katharine Bradley, Libby O. Christensen, Kate Munden-Dixon

**Affiliations:** a Department of Human Ecology, University of California, One Shields Ave., 1309 Hart Hall, Davis, CA 95616, USA; b Independent Scholar, New York, NY, USA; c Colorado State University Extension, 136 6th Street, PO Box 772830, Steamboat Springs, CO, 80477-2830, USA; d Geography Graduate Group, University of California, One Shields Ave., Davis, CA 95616, USA

## Abstract

This article is a description of data related to the research article entitled “The (un)making of ‘CSA people’: member retention and the customization paradox in Community Supported Agriculture (CSA) in California” (Galt et al., in press). The data presented were collected through two statewide surveys, conducted via internet-based questionnaire, related to Community Supported Agriculture in California: a former CSA member survey, and a current CSA member survey. We gathered responses for these surveys from April 2014 to January 2015. The data include responses from 409 former CSA members (those who had left) from 27 CSAs and 1149 current CSA members from 41 CSAs. The data tables included here contain information relevant to the retention of CSA members and other concerns, and come from two analyses: 1) comparisons of characteristics of former and current CSA members, and 2) importance-satisfaction analysis (ISA) of former and current CSA members’ experiences with CSA. We make the detailed results of these analyses available in this article so they can inform other researchers’ analyses of the increasingly important phenomenon of CSA member retention, and, more generally, customers’ participation in and satisfaction with a variety of alternative food networks (AFNs).

**Specifications table**

**Table:** 

Subject area	*Alternative Food Networks (AFNs)*
More specific subject area	*Community Supported Agriculture (CSA)*
Type of data	*Tables*
How data was acquired	*Internet-based questionnaires*
Data format	*Analyzed*
Experimental factors	*CSA farmers were asked to share surveys of former and current members with their membership*
Experimental features	*Data were gathered from a survey of two populations: former CSA members (those who had discontinued membership) and current CSA members*
Data source location	*California*
Data accessibility	*Analyzed data tables are included with this article*
Related research article	*Ryan E. Galt, Katharine Bradley, Libby O. Christensen, and Kate Munden-Dixon. The (un)making of “CSA people”: Member retention and the customization paradox in Community Supported Agriculture (CSA) in California.* Journal of Rural Studies*, in press*[1]

**Value of the data**

• The data provide detailed comparisons of current CSA members with former CSA members in areas that might affect continued membership — enjoyment of food-related activities, conditions interfering with CSA participation, household and individual demographics, and use of food access programs (Table 1, Table 2, Table 3, Table 4) — and are valuable because they provide a fairly comprehensive way of examining variables possibly relevant to continued CSA membership, an important part of CSA member retention.

**Table 1:** Enjoyment of food-related activities, former and current members^.

	Former members			Current members			*t*-tests^ ^	
	mean	st. dev.	*n*	mean	st. dev.	*n*	*t*	*p*
Cooking and food preparation	4.4	0.8	404	4.6	0.6	1145	5.24	0.00***
Learning about cooking, food preparation, and/or preserving	4.3	0.8	402	4.5	0.7	1140	3.64	0.00***
Gardening	3.9	1.1	383	4.1	0.9	1100	2.75	0.01**
Preserving food	3.5	0.9	300	3.8	0.8	910	4.32	0.00***
Shopping for food	3.4	1.1	404	3.7	0.9	1141	3.87	0.00***
Fishing, hunting, and/or foraging	2.7	1.3	323	3.4	1.1	887	8.33	0.00***

^ Likert-scale questions, with 5 = greatly enjoy, 4 = enjoy, 3 = neutral, 2 = dislike, 1 = greatly dislike.

^ ^ Two-tailed, unequal variance assumed.

**Table 2:** Conditions interfering with CSA participation, former and current members.

Question: Have any of the following ever interfered with your household׳s CSA participation?										
	Former members			Current members			*t*-tests^		Correlation with *inconvenient to pick up or receive the share*, former members	
	mean	st. dev.	*n*	mean	st. dev.	*n*	*t*	*p*	*r*	*p*
Work schedules	42.7%	0.5	405	28.8%	0.5	1144	−4.95	0.00***	0.28	0.00***
Child care issues	11.6%	0.3	405	5.6%	0.2	1132	−3.48	0.00***	0.16	0.00***
Lack of transportation	12.3%	0.3	405	6.0%	0.2	1129	−3.55	0.00***	0.25	0.00***

^ Two-tailed, unequal variance assumed.

**Table 3:** Demographics of former and current members.

	Former members			Current members				
	*n* = 362 to 398			*n* = 1050 to 1105			*t*-tests^	
	mean	st. dev.	n	mean	st. dev.	n	*t*	*p*
*Household (HH) variables*								
HH size	2.7	1.3	388	2.7	1.3	1094	0.48	0.63
HH average age	37.7	14.9	388	37.5	15.2	1094	−0.21	0.83
HH has children under 15	0.36	0.48	388	0.39	0.49	1097	1.05	0.29
HH is only 65 and up	0.04	0.19	388	0.04	0.21	1094	0.53	0.60
Number of HH members:								
Under age 15	0.6	1.0	388	0.6	0.9	1097	0.22	0.83
Age 15–24	0.2	0.6	388	0.2	0.5	1097	−0.83	0.41
Age 25–34	0.4	0.8	388	0.5	0.9	1097	0.92	0.36
Age 35–44	0.6	0.8	388	0.5	0.8	1097	−0.61	0.54
Age 45–54	0.4	0.7	388	0.4	0.7	1097	0.07	0.94
Age 55–64	0.3	0.6	388	0.3	0.6	1097	−0.01	0.99
Age 65 and up	0.1	0.4	388	0.1	0.4	1097	1.06	0.29
Number of cars owned by HH	1.7	0.8	398	1.7	0.7	1105	0.46	0.65
Number of adults working full time	1.4	0.6	379	1.3	0.8	1054	−2.48	0.01**
Number of adults working part time	0.3	0.5	379	0.4	0.6	1054	2.75	0.01**
Income category^ ^	7.7	2.0	383	7.6	1.8	1050	−1.17	0.24
*Respondent individual variables*								
Education^ ^ ^	8.9	1.4	394	9.2	1.2	1103	3.36	0.00***
Gender is female	0.86	0.35	375	0.87	0.58	1081	0.44	0.66
Latino	0.07	0.25	382	0.05	0.23	1093	−0.80	0.43
White, non-Latino, alone	0.77	0.42	363	0.81	0.39	1061	1.74	0.08
Black, alone	0.01	0.12	362	0.01	0.12	1067	0.03	0.97
Native American, alone	0.00	0.05	362	0.00	0.03	1067	−0.63	0.53
Asian, alone	0.11	0.31	362	0.08	0.27	1067	−1.69	0.09
Hawaiian Native and Pacific Islander, alone	0.00	0.05	362	0.00	0.04	1068	−0.29	0.77

^ Two-tailed, unequal variance assumed.

^ ^ 1 = Less than $10,000, 2 = $10,000 to $14,999, 3 = $15,000 to $24,999, 4 = $25,000 to $34,999, 5 = $35,000 to $49,999, 6 = $50,000 to $74,999, 7 = $75,000 to $99,999, 8 = $100,000 to $149,999, 9 = $150,000 to $199,999, 10 = $200,000 or more

^ ^ ^ 1 = Middle school or less, 2 = Some high school, 3 = High school degree, 4 = Some technical school, 5 = Technical school certificate program, 6 = Some college, 7 = Associates degree, 8 = Bachelors degree, 9 = Some graduate school, 10 = Graduate degree

**Table 4:** Use of food access programs of former and current members.

	Former members			Current members			*t*-tests^	
	mean	st. dev.	*n*	mean	st. dev.	*n*	*t*	*p*
Reduced cost or free school meals	0.01	0.11	400	0.01	0.09	1112	−0.56	0.57
Produce prescription from a doctor or nurse	0.01	0.10	399	0.01	0.09	1110	−0.34	0.74
CalFresh (a.k.a. food stamps, SNAP - Supplemental Nutrition Assistance Program)	0.01	0.10	400	0.01	0.09	1111	−0.17	0.86
WIC - Women, Infants and Children	0.01	0.07	400	0.00	0.07	1112	−0.12	0.90
Farmers Market Nutrition Program	0.00	0.05	400	0.00	0.03	1111	−0.60	0.55
Food bank or food pantry	0.01	0.07	400	0.00	0.05	1112	−0.60	0.55
Soup kitchen or similar meal program	0.00	0.00	400	0.00	0.04	1112	1.41	0.16
Use of any of the above food access support program	0.03	0.16	400	0.02	0.15	1113	−0.54	0.59

^ Two-tailed, unequal variance assumed.
• The data showing a detailed analysis of the gap between former and current members’ ratings of importance of, and satisfaction with, various CSA attributes (Table 5) is valuable because it allows others to replicate what Galt et al. [1] refer to as importance-satisfaction analysis (ISA), which is a modified version of importance-performance analysis (IPA) commonly done in business settings [2], [3].

**Table 5:** Importance of, and satisfaction with, CSA attributes by former^a^ and current members.

***Descriptive statistics***												
	Former members^a^						Current members					
Column labels for calculations and comparisons:	*A*		*B*		*C* = *B*-*A*		*D*		*E*		*F* = *E*-*D*	
	Importance^b^		Satisfaction^c^		Gap (Satisfaction-Importance)		Importance^b^		Satisfaction^c^		Gap (Satisfaction-Importance)	
	mean	*n*	mean	*n*	mean	*n*	mean	*n*	mean	*n*	mean	*n*
Appropriate diversity of products in the share	4.35	373	3.31	370	−1.05	367	4.29	1128	4.30	1128	0.00	1112
Appropriate quantity of food in the share	4.51	375	3.76	373	−0.75	372	4.35	1140	4.57	1141	0.22	1133
Ability to choose share items/content	3.62	360	2.95	322	−0.67	317	2.69	857	3.74	641	0.79	607
High quality produce	4.89	375	4.22	374	−0.67	373	4.92	1141	4.75	1147	−0.16	1139
Affordability	3.95	374	3.42	373	−0.54	371	3.86	1136	4.20	1143	0.33	1132
Convenient pickup/delivery location	4.32	370	3.89	370	−0.44	365	4.32	1133	4.61	1137	0.29	1125
The farm׳s agricultural practices (e.g., organic)	4.36	371	4.23	367	−0.12	362	4.56	1134	4.70	1132	0.13	1119
Health, dietary, &/or lifestyle impacts from membership	3.79	371	4.19	352	0.40	351	3.96	1132	4.56	1126	0.57	1115
Short transportation distances for produce	3.58	374	4.05	366	0.47	364	3.77	1128	4.37	1127	0.58	1110
Ease of communication with CSA staff/farmer	3.24	369	3.84	351	0.61	345	3.58	1132	4.44	1119	0.83	1105
Knowing my farmer personally	2.25	361	3.38	301	1.13	295	2.62	1096	3.86	970	1.12	950
Sense of community in the CSA (incl. member events)	2.21	364	3.40	314	1.20	308	2.55	1108	3.85	1034	1.24	1018
Newsletter	2.28	366	3.62	318	1.34	316	2.58	1104	4.14	1032	1.48	1013

***Comparative statistics***									
	*t*-tests,† Former Members compared to Current Members								
Column labels for calculations and comparisons:	*G* (*A* compared to *D*)			*H* (*B* compared to *E*)			*I* (*C* compared to *F*)		
	Importance			Satisfaction			Gap (Satisfaction-Importance)		
	*t*	*p*††		*t*	*p*††		*t*	*p*††	
Appropriate diversity of products in the share	−1.3	0.2037		16.1	0.0000***		12.9	0.0000***	
Appropriate quantity of food in the share	−3.8	0.0002**		14.8	0.0000***		13.3	0.0000***	
Ability to choose share items/content	−12.6	0.0000***		10.9	0.0000***		14.0	0.0000***	
High quality produce	1.1	0.2849		10.4	0.0000***		9.3	0.0000***	
Affordability	−1.5	0.1353		15.5	0.0000***		10.3	0.0000***	
Convenient pickup/delivery location	−0.1	0.9369		12.0	0.0000***		8.9	0.0000***	
The farm׳s agricultural practices (e.g., organic)	4.0	0.0001***		10.4	0.0000***		4.7	0.0000***	
Health, dietary, &/or lifestyle impacts from membership	2.7	0.0082*		8.6	0.0000***		3.6	0.0004**	
Short transportation distances for produce	3.0	0.0026*		7.0	0.0000***		1.8	0.0691	
Ease of communication with CSA staff/farmer	5.2	0.0000***		11.6	0.0000***		3.8	0.0001**	
Knowing my farmer personally	5.9	0.0000***		9.2	0.0000***		1.8	0.0774	
Sense of community in the CSA (incl. member events)	5.7	0.0000***		9.2	0.0000***		2.0	0.0469	
Newsletter	5.1	0.0000***		10.4	0.0000***		3.0	0.0025*	

a The sample of former members excludes those who left for the completely exogenous reasons (those outside of the CSA-member relationship) (see [1]).

b 5 = important AND essential for continuing my CSA, 3.75 = important BUT NOT essential for continuing my CSA, 2.5 = of minor importance, 1.25 = not important.

c 5 = very satisfied, 4 = satisfied, 3 = neutral/mixed feeling, 2 = unsatisfied, 1 = very unsatisfied.

† Two-tailed, unequal variances assumed.

†† Significance shown as: * *p* ≤ 0.01 , **p ≤ 0.001, ***p ≤ 0.0000 (these are more conservative since the t-tests are particularly sensitive here).
• Both of the above analyses can be applied to better understand consumers involved and formerly involved in other CSAs or other forms of alternative food networks (ANFs) (e.g., farmers’ market shoppers, you-pick consumers, etc.), thereby allowing better understanding of the populations that continue to engage with a particular kind of AFN compared to those that have discontinued their engagement with that AFN.

## 1 Data

The data that compares various characteristics of former and current CSA members are presented in Table 1, Table 2, Table 3, Table 4, Table 5. Table 1, Table 2 present data that is significantly different between current and former members, specifically about enjoyment of food-related activities (Table 1) and conditions interfering with CSA participation (Table 2). Table 3, Table 4 present data where few to no significant differences were found: demographics (Table 3, see [1] for a discussion of similarities and differences), and food access programs (Table 4). Table 5 presents the analyzed numerical data for the importance-satisfaction analysis (ISA) conducted by Galt et al. [1].

## 2 Experimental design, materials and methods

The data in Table 1, Table 2, Table 3, Table 4 were gathered through statewide surveys of former and current CSA members. We created Internet-based questionnaires for former and current members, one survey for each group, with most questions shared, but with some disparate sections (e.g., reasons for leaving for former members). We sent the two questionnaire links to CSA farmers in California so they could share them with their members. Both surveys were open between April 2014 and January 2015, with numerous reminder emails sent to farmers to remind their former and current members to respond. For former members, the survey collected 409 complete responses from 27 CSAs (for details, see [1]). For current members, the survey collected complete responses for 1149 from 41 CSAs (for details, see [4]). We compared a large number of variables between the two populations using t-tests, and present many of these comparisons thematically in Table 1, Table 2, Table 3, Table 4.

The data in Table 5 is the numerical data behind the importance-satisfaction analysis (the visualization of the data appears in [1]). The analysis compares former and current CSA members’ ratings of importance of, and satisfaction with, various CSA attributes, and the gap between them (for methodological details, see [1]). Unlike other studies of former CSA members, we removed the former members whose membership ended due to reasons completely exogenous to the member-CSA relationship, because these reasons are completely unrelated to CSA management choices (i.e., a CSA ending operation or closing a drop-off area, members moving out of the area, and members experiencing large changes to their household situations, like finances). Since CSA management cannot influence these former members who left for completely exogenous reasons, we argue it is best to remove them from this analysis and other analyses that seek to understand why former members voluntarily leave CSA (see also [1]).
